# Integrated machine learning, molecular dynamics, and density functional theory approaches for identifying potential inhibitor targeting TEM 𝛽-lactamase in *Escherichia coli*

**DOI:** 10.3389/fbinf.2026.1840806

**Published:** 2026-05-20

**Authors:** Aditi Roy, Anand Anbarasu

**Affiliations:** 1 Medical and Biological Computing Laboratory, School of Bio-Sciences and Technology (SBST), Vellore Institute of Technology (VIT), Vellore, Tamil Nadu, India; 2 Department of Biotechnology, School of Bio-Sciences and Technology (SBST), Vellore Institute of Technology (VIT), Vellore, Tamil Nadu, India

**Keywords:** extended-spectrum 𝛽-lactamases, *in silico*, intermolecular interactions, molecular docking, resistance, virtual screening

## Abstract

Enzyme-mediated antimicrobial resistance via *β*-lactamases, including TEM-1 and TEM-235 in *Escherichia coli*, significantly undermines the therapeutic efficacy of *β*-lactam antibiotics. *β*-lactamases are primary drivers of resistance against *β*-lactam drugs and remain critical targets for inhibitor development. In this study, 3576 antibacterial compounds were evaluated for drug-like pharmacokinetic properties, of which 55 compounds that met drug-likeness and non-toxicity criteria were shortlisted for virtual screening against TEM *β*-lactamase. Molecular docking analysis showed that compound 344,265 exhibited high binding affinities with both TEM-1 (−8.5 kcal/mol) and TEM-235 (−8.4 kcal/mol), which are primarily determined by the H-bonding and multiple intermolecular interactions. Stable protein-ligand interactions were demonstrated using molecular dynamics simulations and binding energy calculation using molecular mechanics/Poisson-Boltzmann surface area method. The density functional theory analysis showed that the compound had a moderate HOMO-LUMO energy gap, indicating chemical stability. Also, the surface mapping of the molecular electrostatic potential revealed important reactive sites that could be used to achieve favorable binding orientations. These results demonstrate compound 344,265 as a promising lead molecule targeting the TEM enzyme. Still, it needs further experimental validation to confirm its inhibitory activity and translate these *in silico* findings into therapeutic applications.

## 1 Introduction

Antimicrobial resistance (AMR) is a major public health issue in the 21st century, and *Escherichia coli* (*E. coli*) is among the most common multidrug-resistant (MDR) bacterial pathogens (Paterson and Bonomo, 2005; Miryala and Ramaiah, 2019; Mousa et al., 2025). The extensive use of *β*-lactam antibiotics has exerted considerable selective pressure, and *β*-lactamases have evolved and diversified, thereby weakening the effectiveness of these antibiotics (Baishya et al., 2021). As a result, *β*-lactamase-producing strains are associated with a longer hospital stay, greater treatment costs, and an increased mortality rate, which highlights the immediate need to design new inhibitory measures (Paterson and Bonomo, 2005).

Based on sequence similarity, *β*-lactamases are classified into 4 groups: A to D. Classes A, C, and D represent serine *β*-lactamases that use a catalytic serine residue for nucleophilic attack, while class B is a metalloenzyme that hydrolyses the *β*-lactam ring in a zinc-dependent manner. Class A *β*-lactamases efficiently hydrolyze a broad spectrum of antibiotics, including penicillins, cephalosporins, and carbapenems (Bush and Jacoby, 2010). *β*-Lactam antibiotics hinder bacterial proliferation by acylating the active-site serine residue of essential penicillin-binding proteins (PBPs). Consequently, these proteins are unable to catalyze the transpeptidation required for cross-linking of peptide chains, thereby impairing peptidoglycan biosynthesis in the bacterial cell wall (Sauvage et al., 2008). The production of extended-spectrum *β*-lactamases (ESBLs) by Gram-negative bacteria is a major contributor to resistance against broad-spectrum *β*-lactam antibiotics (Paterson and Bonomo, 2005; Pitout and Laupland, 2008). Class A enzymes are encoded by plasmids and are disseminated widely, resulting from the misuse and overuse of *β*-lactams (Paterson and Bonomo, 2005).

Among the *β*-lactamase family, TEM-1 represents one of the most prevalent enzymes found in *E. coli* and other Gram-negative pathogens (Bradford, 2001). Since its discovery in the 1960s, it has spread worldwide via plasmid-mediated transfer and conferred resistance to penicillins and 1st-generation cephalosporins (Bradford, 2001; Gehlot and Hariprasad, 2022). The structural flexibility of TEMs, together with persistent antibiotic selective pressure, has led to the emergence of numerous TEM variants, many of which exhibit extended-spectrum or inhibitor-resistance (Bush, 2018). Under continuous antibiotic selective pressure, variants, namely, TEM-235, have emerged, highlighting the evolutionary adaptability of TEM enzymes (Imtiaz et al., 1993; Fonzé et al., 1995; Wang et al., 2003; Gray et al., 2025). Therefore, a detailed understanding of the molecular characteristics of TEM-1 and TEM-235 is essential for designing inhibitors that can restore the effectiveness of *β*-lactams.

Residue Ser235 is located within the conserved *β*3 strand of class A TEM *β*-lactamases and helps shape the active-site architecture and substrate-binding pocket. This region plays an important role in determining substrate specificity and inhibitor recognition (Imtiaz et al., 1993; Rudgers and Palzkill, 1999). Substitution of serine with alanine at position 235 (S235A) removes the polar hydroxyl group, resulting in altered hydrogen-bonding capacity and local electrostatic environment (Fonzé et al., 1995). Such changes can influence ligand accommodation and binding interactions within the active site. Previous structural and mechanistic studies have highlighted the importance of residues within this region, including Ser235, in maintaining proper active-site geometry and facilitating interactions with *β*-lactam substrates and inhibitors (Rudgers and Palzkill, 1999).

Mutations in and around the active-site regions of TEM *β*-lactamases have been associated with altered catalytic efficiency and functional adaptability, contributing to the evolution of antibiotic resistance (De Wals et al., 2009). Therefore, the S235A mutation provides a relevant model for investigating how localized structural perturbations can affect ligand binding and inhibitory potential in TEM *β*-lactamases.

To enhance the *β*-lactam efficacy and prevent the emergence of antibiotic resistance, *β*-lactamase inhibitors are prioritized. These inhibitors can function either as suicide inhibitors or as competitive inhibitors through secondary reactions occurring at the active site, irreversibly inactivating the enzyme. The first effective *β*-lactamase inhibitor, clavulanic acid, was identified in 1976, which was co-administered with ticarcillin and amoxicillin. Two synthetic penicillin-derived sulfone *β*-lactamase inhibitors, sulbactam and tazobactam, were subsequently formulated and coupled with the antibiotics piperacillin and ampicillin to enhance their activity. However, the evolution of resistance to *β*-lactam/*β*-lactamase inhibitor combinations has been facilitated by the selective pressure imposed by antibiotic overuse (Drawz and Bonomo, 2010). Clavulanic acid causes mild gastrointestinal problems, including nausea, vomiting, abdominal pain, and loose motion. Antibiotic-associated diarrhea is the most frequent side effect among patients receiving amoxicillin-clavulanic treatment (Evans, 2024). Similarly, although the ceftazidime-avibactam combination demonstrates significant clinical efficacy, its use has been associated with adverse events, including nausea, diarrhea, and headache, which may affect treatment tolerability in certain patients (Papp-Wallace et al., 2011; Cheng et al., 2020; Torres et al., 2023). These limitations highlight the need to identify and develop novel *β*-lactamase inhibitors with improved efficacy and safety profiles.

Compounds with reported antibacterial activity against *E*. *coli* represent promising candidates for identifying molecules that target bacterial resistance mechanisms. Since TEM *β*-lactamases play a central role in conferring *β*-lactam resistance in *E. coli*, molecules active against this pathogen may interact with and inhibit these enzymes (Bradford, 2001). Therefore, exploring antibacterial compounds provides a rational strategy for identifying new inhibitors that could help restore the efficacy of *β*-lactam antibiotics.

**FIGURE 1:**
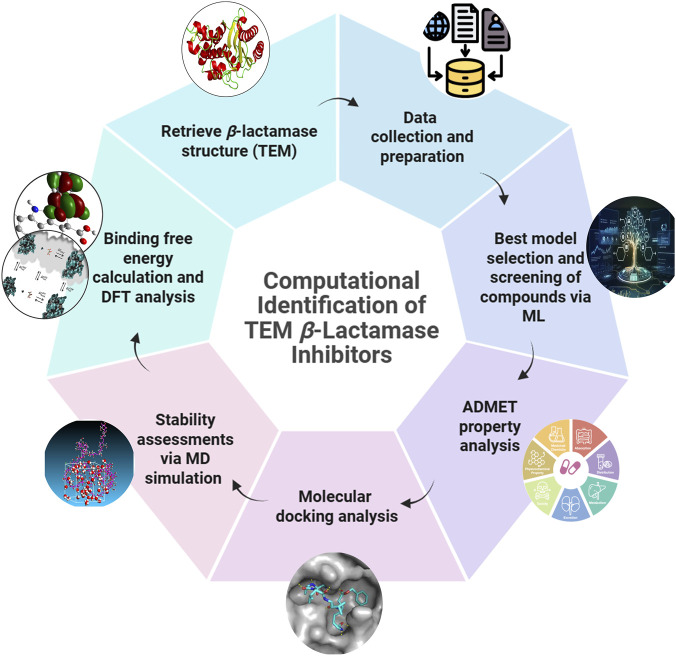
Schematic representation of the virtual screening pipeline employed to identify effective *β*-lactamase inhibitors.

In this study, we aimed to identify a promising TEM inhibitor by screening the PubChem database. Here, 4,681 compounds were screened and filtered for specific pharmacokinetic properties. After molecular docking, the compounds were filtered based on binding energy, and molecular dynamics (MD) simulations, along with other analyses, were conducted to validate the structural stability of the complexes, as shown in Figure 1.

## 2 Materials and methods

### 2.1 Protein structure preparation and optimization

RCSB-PDB (PDB ID:1ERO) provides the 3D structure of TEM-1 from *E. coli.* X-ray crystallography was used to determine the structure of TEM-1 with a resolution of 2.10 Å. PyMOL was used to remove the heteroatoms and water molecules from the original structure. Serine residue at position 235 in TEM-1 was substituted with alanine using SPDBV to generate TEM-235. Global energy minimization of the entire protein structure was performed using SPDBV with the GROMOS 43B1 force field (Kaplan, 2001). The energy-minimized structure was then used in further analyses.

Although a TEM-235 structure is available (PDB ID: 1ESU) it contains missing residues at positions 57, 239, and 253. To minimize structural inconsistencies arising from loop reconstruction, the S235A substitution was incorporated into the complete TEM-1 template (PDB ID: 1ERO), ensuring structural continuity for subsequent analyses. Structural alignment and root-mean-square deviation (RMSD) calculations were performed using PyMOL by superimposing the modeled TEM-235 structure onto 1ESU using backbone Cα atoms, yielding a Cα RMSD of 0.275 Å over 263 aligned residues. This indicates high structural similarity and supports the validity of the modeled structure for subsequent analyses.

### 2.2 Retrieval and preparation of ligands

The compounds with activity against TEM *β*-lactamase were retrieved from the ChEMBL (CHEMBL 2065) database (Zdrazil et al., 2024). Overall, 155 compounds targeting TEM were used for model development and validation, with pIC50 values calculated as the negative base-10 logarithm of IC50. Compounds were categorized as active if their pIC50 values were greater than 6 and as inactive if they were less than 6. For the test dataset, 4,681 antibacterial compounds were retrieved from the PubChem database (Kim et al., 2021). The three-dimensional (3D) structures of all compounds were generated from their canonical SMILES using Open Babel v3.1.1, followed by geometry optimization employing the MMFF94 force field to obtain energetically favorable conformations (O’Boyle et al., 2011). The structural quality of the optimized conformers was assessed based on energy minimization convergence, removal of steric clashes, and chemically valid bond geometries. Duplicate structures and molecules with incomplete or inconsistent valence states were excluded. Furthermore, the lowest-energy conformer for each compound was selected for downstream descriptor calculation and virtual screening to ensure structural consistency across the dataset. Additionally, protonation states were implicitly considered under physiological pH, and all structures were converted to standardized SDF format to maintain compatibility with descriptor calculation tools.

Although the machine learning model was trained on a relatively smaller curated dataset (n = 155), this dataset comprised experimentally validated bioactivity values (pIC50) obtained from ChEMBL, ensuring high data reliability (Zdrazil et al., 2024). The model was rigorously evaluated using 10-fold cross-validation along with multiple statistical metrics, including ROC, kappa statistics, sensitivity, and specificity, to minimize the risk of overfitting and ensure predictive robustness (Colakoglu et al., 2019). The larger dataset of 4,681 compounds retrieved from PubChem was not used for model training but served as an independent external screening set, which is a standard practice in virtual screening workflows. Therefore, the difference in dataset sizes reflects the transition from model development to large-scale screening rather than overfitting. The model’s ability to identify potential hits from a chemically diverse external dataset further supports its generalizability.

### 2.3 Molecular descriptors calculation and preprocessing of the dataset

Chemical descriptors characterize the structural and physicochemical properties of molecules that influence their biological activity. All the compounds were converted to 3D SDF format using Open Babel v3.1.1 and subsequently imported into PaDEL to calculate chemical descriptors (O’Boyle et al., 2011; Yap, 2011). Overall, 881 molecular descriptors were generated to comprehensively represent the compounds’ structural features for subsequent analysis. The complete data set was processed using the WEKA software to develop a machine learning (ML) model (Asha Kiranmai and Jaya Laxmi, 2018). In order to select features, different WEKA functions were taken into consideration, namely, correlation analysis, attribute evaluation, and missing value handling. The model was constructed using 10-fold cross-validation (CV) (Hall et al., 2009). Random Forest, J48, and Partial Decision Tree (PART), 3 different classifiers, were implemented in WEKA separately to determine the best screening model. During a 10-fold CV, the training dataset will be divided into 10 different sets (Nand et al., 2020). The performance of each model was evaluated using evaluation metrics and confusion matrices, including sensitivity, specificity, Kappa statistics, and accuracy. To assess model accuracy, the following formulas were used.

$$\text{Binary classification accuracy}=\frac{\text{TN}+\text{TP}}{\left(\text{TN}+\text{TP}+\text{FN}+\text{FP}\right)}$$

$$\text{Sensitivity}=\frac{\text{TP}}{\left(\text{FN}+\text{TP}\right)}$$

$$\text{Specificity}=\frac{\text{TN}}{\left(\text{FP}+\text{TN}\right)}$$

### 2.4 Physicochemical property assessment

Virtual screening is useful for identifying potent compounds that bind to target proteins and modulate their activity. Swiss ADME was utilised to evaluate these compounds based on desirable pharmacological properties (Daina et al., 2017). Selection criteria included: MW < 500 Da, <5 H-bonds, <10 H-bond acceptors, <5 logP, and <10 rotatable H-bonds. Topological Polar Surface Area (TPSA) represents the bioavailability of a drug molecule, and the compounds with TPSA below 140 Å^2^ tend to be highly absorbed through the intestine (Lipinski et al., 2012). ProTox 3.0 was used to perform toxicological assessment of the screened compounds; it considers four key categories, including hepatotoxicity, cardiotoxicity, carcinogenicity, and mutagenicity (Banerjee et al., 2024). Only non-toxic substances with an LD50 ≥ 1000 mg/kg were selected for further investigation.

### 2.5 Protein-ligand docking analysis

AutoDock Vina was used to dock all the filtered molecules with the protein to investigate intermolecular interactions (Eberhardt et al., 2021). PrankWeb predicted the active site, and the coordinates were obtained using PyMOL (Polák et al., 2025). The active site coordinates were X = 40.878, Y = 36.806, and Z = 32.541, centered within a grid box of 25 × 25 × 25 Å^3^. A default exhaustiveness value of 8 and an energy range of 4 kcal/mol were used in experimental molecular docking to guarantee computational performance. The purpose of docking was to examine how the screened small molecules bound to the proteins (Meng et al., 2011; Kumar et al., 2014; Malathi et al., 2016). For further examination, the compounds with lower binding energies were considered. 2D and 3D conformers of the best-docked complexes were visualized using BIOVIA Discovery Studio Visualizer and UCSF ChimeraX, respectively (Pettersen et al., 2021).

### 2.6 Stability assessment using MD simulation

MD simulation using GROMACS 2024.2. Was used to assess the stability and dynamics of the docked complexes. Ligand topologies were created with the CGenFF server, while the protein topology files were prepared using the CHARMM36 FF (Vanommeslaeghe et al., 2012). A dodecahedral box with a constant boundary distance of 1.2 nm was used to solvate the complexes. To neutralize the system, chloride (Cl^−^) and sodium (Na^+^) ions were added to the solvated models of the docked complexes and proteins (Roy and Anbarasu, 2025). System energy minimization was performed for 50,000 steps, applying a convergence criterion of 10 kJ/mol. System equilibrium was reached in 2 phases: NVT and NPT, each for 100ps. Using a 2 fs time step over a 100 ns time frame, the simulations were performed under physiological parameters at a constant temperature of 300 K and a constant pressure of 1 atm (Biswas and Anbarasu, 2025). The dynamic stability of each complex was analyzed using trajectory analysis. RMSD, solvent-accessible surface area (SASA), radius of gyration (Rg), H-bond interactions, root-mean-square fluctuation (RMSF), and interaction energy were the main variables considered. The analysis provided insight into the stability and dynamics of interactions among all docked complexes through simulation.

### 2.7 Binding free energy calculation

The protein-ligand complex’s binding free energy was calculated using the Gmx_mmpbsa tool in accordance with the MM/PBSA method (Kollman et al., 2000). This experiment provided a detailed assessment of the ligand’s binding affinity, showing maximum binding to both the TEM-1 and TEM-235 forms, and supports the MD simulation results. Using the MD simulation trajectory and the gmx_MMPBSA tool, the binding free energy of each complex was calculated (Miller et al., 2012). The MM/PBSA analysis offered valuable insights into ligand-binding energetics, revealing variations in complex free energies and aiding understanding of ligand interaction dynamics. The graphical representation of the energy components of MM/PBSA was obtained using the gmx_MMPBSA_ana tool (Kumari et al., 2014).

### 2.8 Principal component analysis (PCA)

The PCA was used to analyze the metastable conformational spaces surrounding the native states of proteins in MD simulation trajectories. The magnitude and direction of motion are represented by eigenvalues and eigenvectors, respectively. PCA was conducted using covariance matrices (CMs) generated from the atomic coordinates of protein Cα atoms, thereby minimizing statistical noise. Variations in the Cα atoms of each residue are considered in the covariance matrix, generating orthogonal eigenvectors with their associated eigenvalues.

PCA was carried out using the GROMACS analysis suite. The covariance matrix was constructed with the help of ‘*gmx covar,*’ and principal component trajectory overlap was assessed with “*gmx anaeig*.” (Abdi and Williams, 2010).

### 2.9 Free energy landscape (FEL)

FEL, together with the associated Gibbs free energy, shows all the possible conformations that a protein can adopt during MD simulations. It illustrates two key variables: the first two principal components derived from PCA, which reveal system properties and determine spatial diversity. The plots were generated utilizing “*gmx sham*” script using the acquired PCA values (Papaleo et al., 2009).

### 2.10 Dynamic cross-correlation matrix (DCCM) analysis

DCCM analysis was used to evaluate time-dependent changes in protein Cα atoms induced by ligand binding. For every Cα atom, a correlation matrix was generated, enabling a thorough analysis of inter-domain interactions (Nilewar et al., 2026).

### 2.11 Density functional theory (DFT) analysis

DFT, a quantum approach, was employed to investigate ground-state properties, electronic structure, and atomic-level properties of molecular and material systems (Baerends and Gritsenko, 1997). DFT analysis was used to assess the stability and chemical reactivity of the lead compound. The Becke 3-parameter hybrid exchange functional (B3) combined with the Lee-Yang-Parr (LYP) correlation functional and the 6–311++G (d,p) basis set was used for molecular geometry optimization, electron density mapping, and frontier molecular orbital (HOMO-LUMO) analyses to identify the lowest-energy conformations in Gaussian 16W software and visualize them in GaussView v6.1 software (Dennington, 2016; Karakaş sarikaya et al., 2021). The frontier molecular orbital (FMO) was used to compute the chemical reactivity characteristics. FMO energies, including HOMO–LUMO levels, were assessed in this study together with other electronic descriptors, including electron affinity (EA), electronegativity (χ), chemical potential (µ), electrophilicity index (ω), chemical hardness (η), and softness (S) (Akbari et al., 2024).

## 3 Results

### 3.1 Evaluation of various ML classifiers

**FIGURE 2:**
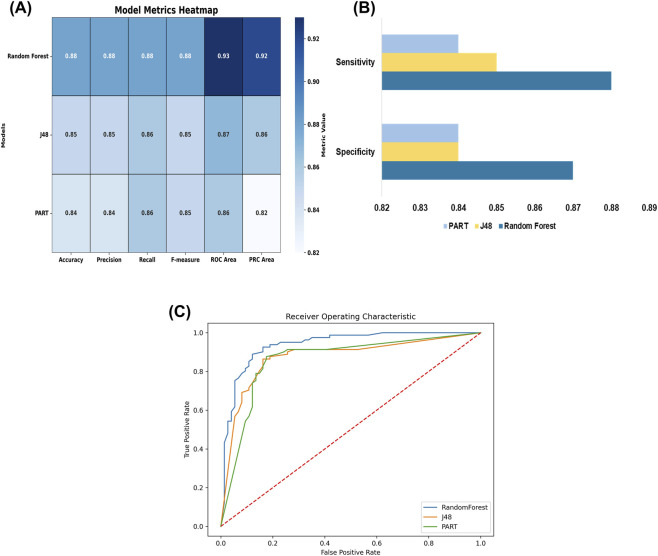
Statistical evaluation of classifier performance for machine learning model development using the training dataset. **(A)** Various model metrices are presented in heatmap, **(B)** Sensitivity and specificity of models, **(C)** ROC curve analysis of various machine learning classifiers.

To differentiate anti-TEM compounds from other inactive compounds, we employed ML methods to develop a classification model. The model performance was evaluated using distinct metrics, as listed in Table 1. A set of models was trained in the training dataset using 3 different classifiers, namely, Random Forest, J48, and PART, employing 10-fold CV. During model evaluation, the kappa statistic measures the agreement between true and predicted classes, where a value of 1 indicates perfect agreement between the ground truth and the classification model. The Random Forest model has the highest kappa statistic, 0.76, and an RMSE of 0.31. The comparison indicated that the best classifier was Random Forest, then J48 and PART. Random Forest, J48, and PART models attained classification accuracies of 88.38%, 85.16%, and 84.51%, respectively, as shown in Figure 2A. Sensitivity and specificity analyses were used to select the best model for each dataset and assess the classifier’s performance in correctly recognizing positive and negative instances, as depicted in Figure 2B. The sensitivity was between 84% and 88% and the specificity was between 84% and 87%. The Random Forest model also had the best sensitivity on the dataset, whereas PART had the lowest. Additionally, performance metrics, including receiver operating characteristic (ROC) curve analysis, were used to assess and validate the models’ strength. The ROC curve, plotted over a range of discrimination thresholds, was used to assess the performance of the binary classification model. The ROC curve initially showed strong proximity to the TP rate axis, indicating a high TP rate and a low FP rate, thereby reflecting optimized sensitivity and specificity. The ROC values for Random Forest, J48, and PART were 0.93, 0.87, and 0.86, respectively, as shown in Figure 2C. Random Forest was chosen as the best-performing model and applied for compound screening. From a set of 4,681 compounds, the model predicted 3,576 as active and selected them for further evaluation.

**TABLE 1:** Comparative evaluation of classifier performance for developing a screening model using the training dataset.

Classifier name	Precision	Correctly classified instances % (value)	Kappa statistic	Mean absolute error	Root mean square error	MCC	ROC	Specificity	Sensitivity
Random forest	0.88	88.38	0.76	0.19	0.31	0.76	0.93	0.87	0.88
J48	0.85	85.16	0.7	0.18	0.36	0.70	0.87	0.84	0.85
PART	0.84	84.51	0.68	0.17	0.37	0.69	0.86	0.84	0.84

### 3.2 ADMET analysis

The chemical structures of 3,576 antibacterial compounds were retrieved from the PubChem database based on their reported antimicrobial activity against *E. coli* for virtual screening (Supplementary Table S1). After pharmacokinetic evaluation using SwissADME, 380 compounds were shortlisted based on factors such as molecular weight, hydrogen-bond donors and acceptors, total polar surface area, gastrointestinal absorption, compliance with Lipinski’s rule of five, and synthetic accessibility (Supplementary Table S2). Subsequently, the selected compounds were subjected to toxicity assessment, including predictions of hepatotoxicity, cardiotoxicity, carcinogenicity, and mutagenicity. Following toxicity evaluation, 55 of them were identified as non-toxic and retained for further analysis (Supplementary Table S3).

### 3.3 Interaction profile of protein-ligand complex

Site-specific intermolecular docking has been performed to assess binding interactions of selected compounds with TEM-1 and TEM-235, using PyRx integrated with AutoDock Vina. This gives us an understanding of the atomic-level molecular mechanism that determines the activity of the compound. This was done to prioritize the possible inhibitors based on their expected binding affinity. Compounds with high docking scores in comparison with the reference compound, avibactam, were shortlisted as promising compounds to proceed further (Supplementary Table S4). The docking score of each ligand against each variant of TEM-1 and TEM-235 was calculated to assess the relative binding potential of compounds. Out of the compounds screened, 344265, 2083510, and 10543588 showed the best binding affinity with both TEM-1 and TEM-235 variants. Against TEM-1, the docking scores −8.5, −8.3, and −8.2 kcal/mol, whereas against TEM-235, the docking scores were −8.4, −8.3, and −8.1 kcal/mol (Table 2). Avibactam, the reference inhibitor, had a docking score of −6.7 kcal/mol against TEM-1 and -6.5 kcal/mol against TEM-235.

**TABLE 2:** Molecular interaction profiles of the screened compounds.

TEM variants	Ligand	Binding affinity (kcal/mol)	H-bond	Other interactions
TEM-1	344,265	−8.5	Ser130, Val216, Lys234, Ala237	Met69, Ser70, Tyr105, Asn132, Glu166, Asn170, Ser235, Gly236, Arg243
	2,083,510	−8.3	Ser130, Val216, Lys234, Ser235, Arg243	Ser70, Lys73, Glu104, Tyr105, Asn132, Pro167, Asn170, Ala 217, Leu220, Gly236, Ala237, Gly238
	10,543,588	−8.2	Glu104, Pro167, Asn170, Ala237, Glu239	Met69, Ser70, Ser73, Tyr105, Asn132, Glu166, Glu168, Leu169, Glu171, Gly238
	Avibactam	−6.7	Ser70, Ser130, Asn132, Lys234, Ala237	Met69, Glu104, Tyr105, Glu166, Pro167, Asn170, Val216, Ser235, Gly236, Arg243
TEM-235	344,265	−8.4	Ser130, Val216, Ala235, Ala237, Arg243	Met69, Ser70, Tyr105, Asn132, Asn170, Pro219, Lys234, Gly236
	2,083,510	−8.3	Glu104, Asn132, Asn170	Ser70, Tyr105, Ser130, Glu166, Pro167, Val216, Ala235, Gly236, Ala237, Gly238, Glu239, arg 243
	10,543,588	−8.1	Ser70, Glu104, Pro167, Asn170, Ala237, Glu239	Met69, Lys73, Tyr105, Asn132, Glu166, Glu168, Glu171, Gly238
	Avibactam	−6.5	Ser70, Ser130, Asn132, Lys234, Ala235, Ala237, Arg243	Met69, Glu104, Tyr105, Glu166, Pro167, Asn170, Val216, Gly236

**FIGURE 3:**
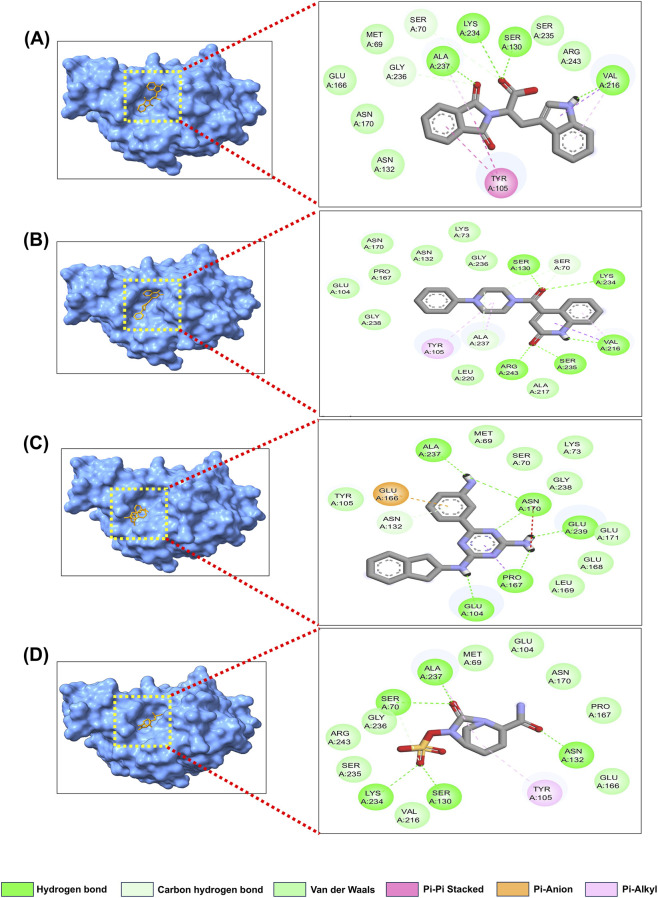
Molecular docking interaction analysis **(A)** TEM-1_344265, **(B)** TEM-1_2083510, **(C)** TEM-1_10543588, **(D)** TEM-1_Avibactam.

**FIGURE 4:**
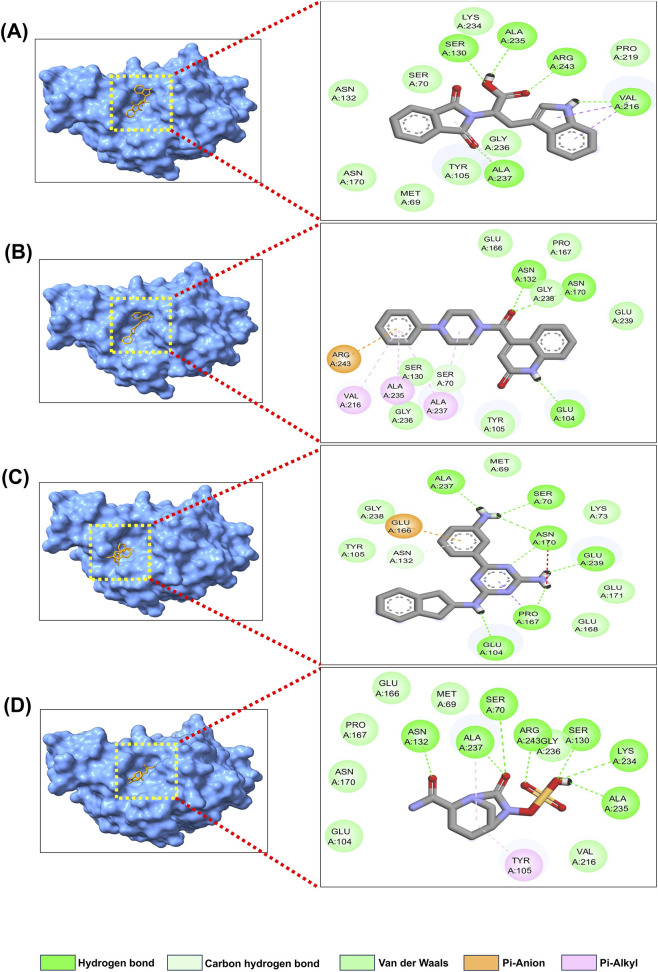
Molecular docking interaction analysis **(A)** TEM-235_344265, **(B)** TEM-235_2083510, **(C)** TEM-235_10543588, **(D)** TEM-235_Avibactam.

The three best candidates were subjected to a detailed molecular docking study to characterize their intermolecular interactions (Table 2; Figure 3). Compound 344265 was found to have a stable binding at the active site of TEM-1 with four H-bonds with major residues Ser130, Val216, Lys234, and Ala237. Further, Met69, Asn132, Glu166, Asn170, Ser235, and Arg243 were found to have six van der Waals interactions. Further stabilizing contacts involved Ser70, Tyr105, Val216, and Gly236 (Figure 3A). The 2083510 showed stable binding to TEM-1, mediated by five hydrogen bonds involving Ser130, Val216, Lys234, Ser235, and Arg243. Additionally, the complex was stabilized by several non-covalent interactions, including van der Waals interactions, C-H bonds, π-σ interactions, alkyl contacts, and π-alkyl contacts. These interactions were observed with Ser70, Lys73, Glu104, Tyr105, Asn132, Pro167, Asn170, Ala217, Leu220, Gly236, Ala237, and Gly238 (Figure 3B). When the TEM-1_10543588 complex was analyzed, 15 distinct intermolecular interactions were identified, consisting of five H-bonds and ten further non-covalent interactions (Figure 3C). TEM-1_avibactam also exhibits 15 intermolecular interactions (Figure 3D). In the active site, five H-bonds and eight van der Waals linkages were identified in the TEM-235_344,265 complex (Figure 4A). The 15 different intermolecular interactions were found in the TEM-235_2083510 complex (Figure 4B), whereas the TEM-235_10543588 complex showed relatively fewer contacts, with 14 interactions identified (Figure 4C). TEM-235_avibactam showed 15 intermolecular interactions as shown in Figure 4D.

Notably, several interacting residues identified in this study, including Ser70, Lys73, Glu166, Ser130, Asn132, and Ala237, are well-established components of the catalytic and substrate-binding pocket of TEM *β*-lactamases. Previous mechanistic studies have reported the critical importance of these residues in the inhibition process. In particular, Ser70 functions as the nucleophilic residue, while Glu166 is involved in catalytic activation through a water-assisted deprotonation mechanism, and Lys73 facilitates proton transfer events associated with Ser70 and Ser130 (Sgrignani et al., 2014).

The involvement of these conserved residues in hydrogen bonding and van der Waals interactions with the screened compounds suggests that the ligands engage key catalytic regions of the enzyme. This indicates that the identified compounds bind within the canonical active site and exhibit interaction patterns consistent with those of known *β*-lactamase inhibitors, such as avibactam, supporting their potential functional and therapeutic relevance.

### 3.4 Evaluation of structural stability of complexes using MD simulations

A 100 ns MD simulation was used to examine the structural stability and dynamic behavior of the three top-ranked compounds and the reference inhibitor avibactam in the presence of TEM-1 and TEM-235. RMSF, RMSD, SASA, hydrogen bonding, and interaction energy (IE) metrics have been used to analyze the trajectories. Among the screened compounds, 344265 demonstrated the most consistent and stable behavior across both TEM variants. It exhibited lower structural deviations (RMSD), reduced residue-level fluctuations (RMSF), favorable interaction energy, and stable hydrogen bonding patterns throughout the simulation. In contrast, the other compounds exhibited greater fluctuations and less consistent binding behavior between the two variants. Therefore, compound 344265 was selected as the most promising inhibitor candidate for further in-depth analysis. The remaining compounds exhibited stable binding with only a single TEM variant and were therefore not included in the primary analysis; their full MD datasets are presented in Supplementary Figure S1 for reference.

#### 3.4.1 Root mean square deviation

RMSD is a standard measure for estimating conformational stability and convergence over the simulation trajectory. It is used to measure the positional change of the backbone Cα atoms relative to a reference structure during the formation of a protein-ligand complex. The reduced RMSD values suggest minimal structural deviation, indicating improved complex strength over time.

**FIGURE 5:**
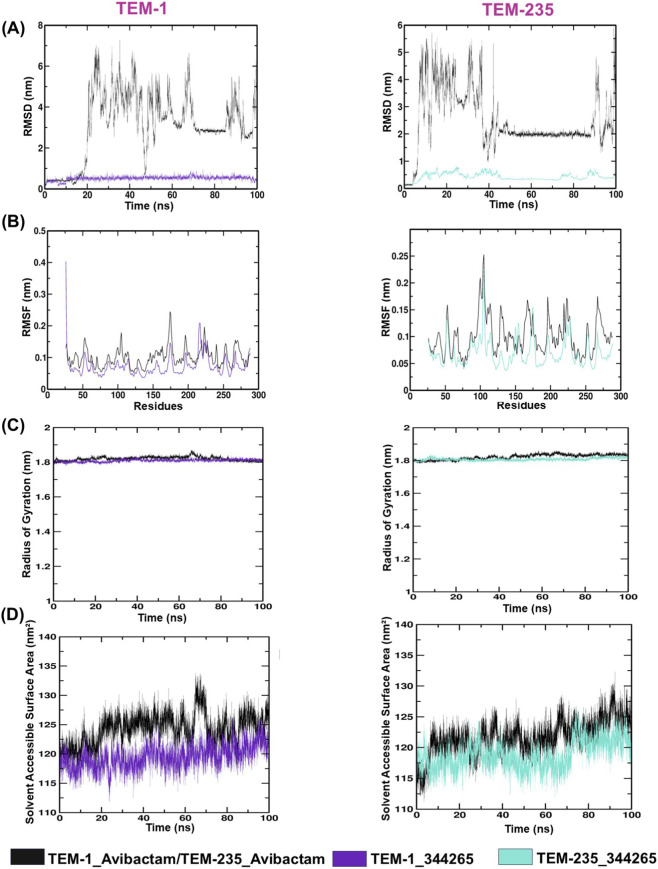
Molecular dynamics simulation analysis of protein-ligand complexes. **(A)** RMSD, **(B)** RMSF, **(C)** Radius of gyration, **(D)** SASA.

The TEM-1_344,265 and TEM-235_344,265 complexes had RMSD of 0.51 and 0.42 nm, respectively, indicating stable conformational behavior. On the other hand, the TEM-1_avibactam and TEM-235_avibactam complexes showed much higher RMSD values of 3.10 and 2.48 nm, respectively, indicating greater structural deviation in the simulation trajectories. TEM-1 344,265 and TEM-235 344,265 retained comparable conformational profiles throughout the simulation, with only slight structural deviations (Figure 5A).

The elevated RMSD values observed for the TEM-avibactam complexes (2.48–3.10 nm) indicate substantial global deviation from the initial structure throughout the simulation. However, RMSD is a global metric that integrates positional changes across all backbone atoms and is therefore highly sensitive to collective structural motions. In systems involving ligand binding, such deviations can arise from conformational adjustments that enable optimal accommodation of the ligand within the binding pocket. These include large-scale rearrangements, local flexibility, and redistribution of structural strain throughout the protein. Consequently, the increased RMSD values are more indicative of ligand-induced conformational changes and reflect the dynamic nature of protein-ligand interactions.

#### 3.4.2 Root mean square fluctuation

To describe residue-specific backbone fluctuations over time within the complexes, RMSF analysis was conducted for all four complexes. The analysis provides specific information on local flexibility patterns and dynamic changes in protein regions during MD simulations. This was determined by RMSF analysis, which indicated average residue changes of 0.07 nm and 0.06 nm for the TEM-1_344,265 and TEM-235_344,265 complexes, respectively. On the other hand, TEM-1_avibactam and the TEM-235_avibactam complexes displayed average fluctuations of 0.10 nm (Figure 5B).

#### 3.4.3 Radius of gyration

Rg was calculated to assess the compactness of the protein and its complexes during the simulations, as well as their folding stability. The mean values of Rg for TEM-1_344,265, TEM-235_344,265, TEM-1_avibactam, and TEM-235_avibactam remained consistently in the range of 1.80–1.82 nm, indicating very slight structural changes during the course of the simulation (Figure 5C). These findings suggest that ligand association did not significantly alter the global protein folding configuration and that all complexes remained compact and structurally stable throughout the MD run.

#### 3.4.4 Solvent accessible surface area

By examining how the protein and solvent interact during ligand binding throughout the MD simulation, SASA provides a quantitative evaluation of protein conformational dynamics and structural stability. The average SASA values for TEM-1_344265, TEM-235_344,265, TEM-1_avibactam, and TEM-235_avibactam was 121.54, 118.78, 124.23, and 121.71 nm^2^, respectively (Figure 5D). The comparable SASA values across the complexes indicate minimal variation in solvent exposure, suggesting that ligand binding does not induce significant conformational changes or destabilization of the TEM protein structure.

#### 3.4.5 Hydrogen bond analysis

**FIGURE 6:**
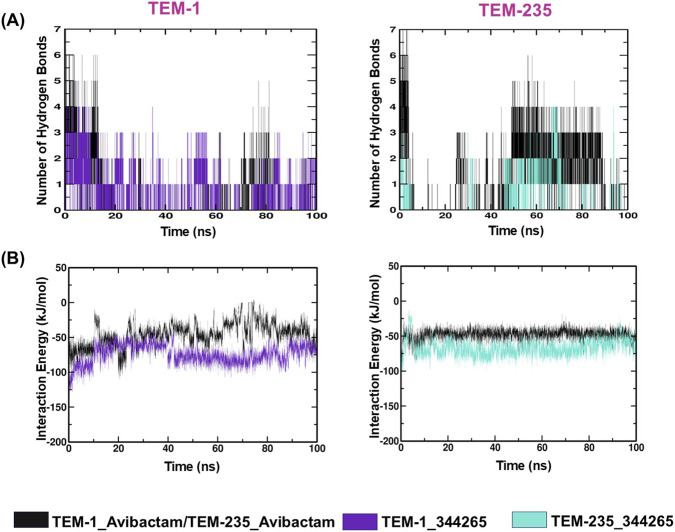
Intermolecular interaction and interaction energy profiles. **(A)** Hydrogen bonds, **(B)** Interaction energy.

H-bonds are vital in the stabilization of ligand protein interactions. The stability of the ligand in the active site is influenced by the establishment and stabilization of H-bonds during simulations. Compound 344,265 formed six hydrogen bonds with TEM-1, one of which sustained throughout the trajectory. With respect to TEM-235, the compound established as many as four hydrogen bonds along the trajectory. Conversely, avibactam was able to establish a maximum of six to seven hydrogen bonds with all of the TEMs; although they were only temporary and were less stable throughout the duration of the simulation (Figure 6A).

#### 3.4.6 Interaction energy

The Parrinello-Rahman algorithm in the GROMACS simulation package was used to calculate the mean LJ-SR interaction energies, representing van der Waals forces. The overall interaction energy was most favorable in the TEM-1_344265 complex (−74.17 kJ/mol), followed by the TEM-235_344265 complex (−66.97 kJ/mol) during the 100 ns simulation period. However, the interaction energies of the TEM-1_avibactam and TEM-235_avibactam complexes were relatively low, at −45.54 and −47.81 kJ/mol, respectively (Figure 6B).

Altogether, compound 344,265 was more strongly bound to both TEM variants across all simulations, confirming the docking findings and indicating a more favorable binding affinity than avibactam.

### 3.5 Binding free energy calculation using MM-PBSA

**FIGURE 7:**
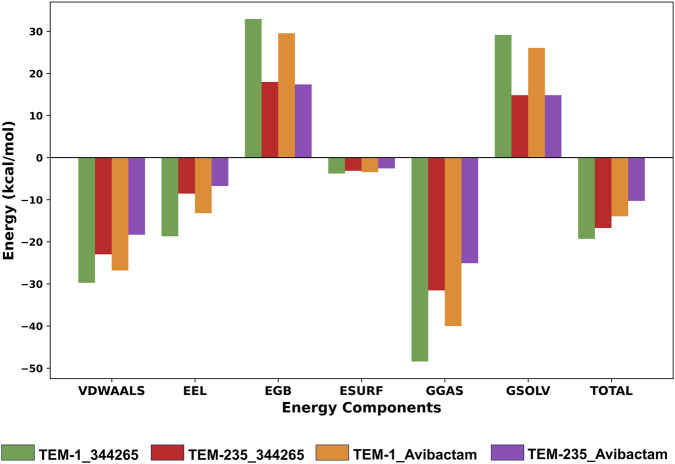
MM/PBSA-based binding free energy calculation with per-residue contributions to the total binding free energy.

Van der Waals molecular mechanics energy (ΔVDWAALS), electrostatic energy (ΔEEL), polar solvation energy (ΔEGB), and SASA energy (ΔESURF) were among the specific components of the total energy that were used to calculate the binding free energy of the complexes (Table 3). These contributions were further added to give total gas-phase energy (ΔGGAS) and total solvation energy (ΔGSOLV), which combined to give the overall binding free energy (ΔGTOTAL). Compound 344,265 exhibited stronger binding affinity toward both TEM-1 and TEM-235, with binding free energies of −19.31 ± 4.08 kcal/mol for TEM-1 and -16.74 ± 8.11 kcal/mol for TEM-235, compared to avibactam, which showed binding energies of −13.96 ± 2.73 kcal/mol for TEM-1 and -10.29 ± 4.77 kcal/mol for TEM-235 (Figure 7).

**TABLE 3:** Binding free energy estimation using MM/PBSA analysis.

Protein-ligand complex	ΔVDWAALS (kcal/mol)	ΔEEL (kcal/mol)	ΔEGB (kcal/mol)	ΔESURF (kcal/mol)	ΔGGAS (kcal/mol)	ΔGSOLV (kcal/mol)	Δtotal (kcal/mol)
TEM-1_344,265	−29.73±3.3	−18.7±6.86	32.9±5.97	−3.79±0.46	−48.43±8.45	29.12±5.7	−19.31±4.08
TEM-235_344,265	−23±9.25	−8.56±10.25	17.97±10.55	−3.15±1.33	−31.56±15.45	14.82±9.86	−16.74±8.11
TEM-1_Avibactam	−26.82±3.45	−13.19±5.28	29.53±5.13	−3.48±0.44	−40.01±6.52	26.05±4.88	−13.96±2.73
TEM-235_Avibactam	−18.34±6.71	−6.75±7.34	17.39±8.14	−2.59±0.92	−25.09±11.49	14.81±7.45	−10.29±4.77

### 3.6 Conformational space analysis

**FIGURE 8:**
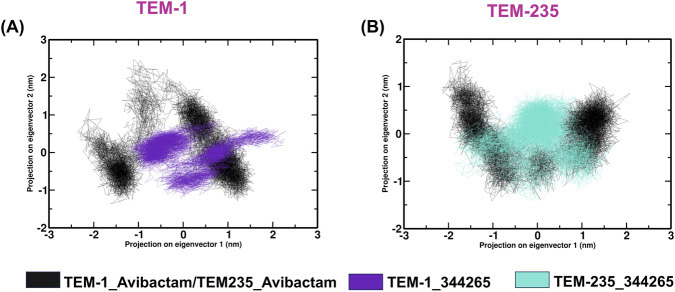
Two-dimensional conformational projection from principal component analysis. **(A)** TEM-1_344265/Avibactam, **(B)** TEM-235_344265/Avibactam.

PCA has been performed to characterize dominant collective motions and conformational transitions of TEM-1 and TEM-235 upon ligand binding during the molecular dynamics simulations. Atomic coordinate fluctuations over the 100 ns trajectory were compiled into a covariance matrix, from which eigenvectors were extracted to describe the principal modes of motion governing trajectory dynamics. The extent of conformational space sampled by the complexes indicates an enhanced structural stability upon inhibitor binding. A 2D projection of the first PCs was generated to visualize the influence of ligand association on TEM dynamics, thereby enabling comparative assessment of the global motion patterns of the complexes (Figure 8).

### 3.7 Thermodynamic energy landscape analysis

**FIGURE 9:**
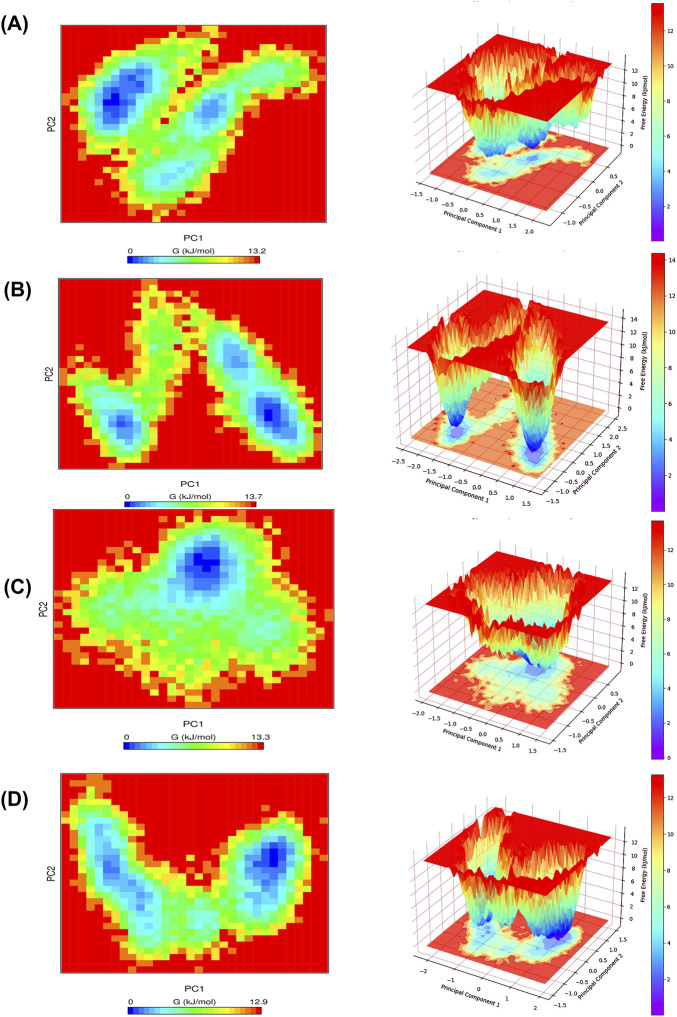
Graphical depiction of the free energy landscape derived from PCA for protein-ligand complexes. **(A)** TEM-1_344265, **(B)** TEM-1_Avibactam, **(C)** TEM-235_344265, **(D)** TEM-235_Avibactam.

FEL analysis was performed to evaluate protein conformational changes based on energy and simulation time. The conformational space defined by the first two PCs, PC1 and PC2, was used to calculate the Gibbs free-energy landscape. In the FEL plots, blue regions signify low-energy, thermodynamically stable conformations, whereas red regions signify higher-energy, less stable states. The calculated Gibbs free energy ranges for TEM-1_344,265, TEM-235_344,265, TEM-1_avibactam, and TEM-235_avibactam were 0–13.2, 0–13.3, 0–13.7, and 0–12.9 kJ/mol, respectively (Figure 9). The comparable and confined energy distributions support the thermodynamic stability of all complexes throughout the simulation period.

### 3.8 Cross-correlation analysis of residue motions

**FIGURE 10:**
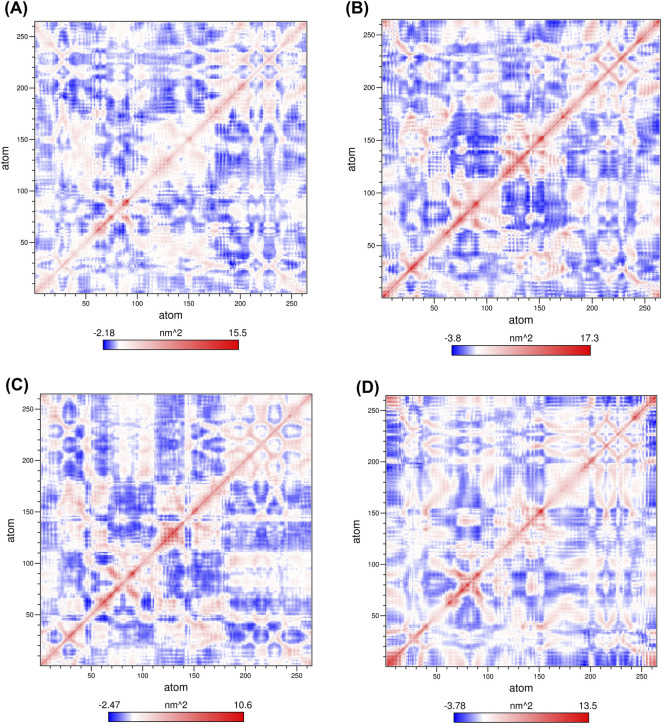
Analysis of cross-correlation profiles for protein-ligand complexes using Cα residues. **(A)** TEM-1_344265, **(B)** TEM-1_Avibactam, **(C)** TEM-235_344265, **(D)** TEM-235_Avibactam.

Using Cα atomic coordinates obtained from the 100 ns MD trajectories, DCCM analysis was performed to examine the influence of inhibitor binding on the internally correlated motions of TEM. The resulting correlation matrices for each protein-ligand complex are presented in Figure 10. These matrices show patterns of residue-level fluctuation of both TEM-1 and TEM-235 and the distribution of positively and negatively correlated motions during the simulation period. The strength and trend of the correlation are shown by the color intensity in the DCCM plots; blue areas indicate a negative correlation (residues move in the opposite direction), and red areas indicate a positive correlation (residues move in the same direction). The correlation coefficients are between −1 and +1; +1 means strongly correlated motion, and −1 means absolutely anti-correlated motion.

The simulations showed correlated and anti-correlated motions at the residue level throughout the analysis. The TEM-1 and TEM-235_avibactam complexes showed widespread areas of anti-correlated motion of the complexes, consisting of several clusters of residues, indicating the presence of strong opposite directional motions and a more flexible protein structure. Conversely, the complexes with compound 344,265 exhibited less anti-correlated fluctuations and correlated motions that were more localized. This tendency indicates that, in the presence of 344,265, the dynamics of residues are better coordinated, and that immensely large-scale, counter-directional motions are also reduced. Overall, the profiles of the DCCM samples show that compound 344,265 is more effective at stabilizing the internal dynamics of both TEM variants than the reference inhibitor avibactam.

### 3.9 DFT calculations

The top-ranked compound 344,265 was analyzed by density functional theory (DFT) to obtain quantitative insight into its electronic structure, energetic stability, and chemical reactivity. The HOMO and LUMO energies reflect the molecule’s electron-donating and electron-accepting potentials, respectively, and provide insight into its potential interactions with biological targets. The HOMO–LUMO energy gap (ΔE) is a crucial descriptor of chemical reactivity and kinetic stability. The HOMO and LUMO energies of compound 344,265 were calculated as −5.76 eV and −2.71 eV, respectively, resulting in an energy gap of 3.05 eV.

The magnitude of the HOMO–LUMO gap reflects the balance between molecular stability and reactivity. In general, a lower energy gap is associated with higher chemical reactivity, whereas a higher energy gap indicates greater kinetic stability and lower intrinsic reactivity. The observed energy gap of 3.05 eV suggests that compound 344,265 maintains a favorable equilibrium between stability and reactivity, which is desirable for effective interaction with biological targets.

The reference inhibitor avibactam exhibits a higher HOMO–LUMO energy gap (5.49 eV), reflecting greater intrinsic stability. In contrast, the comparatively lower energy gap of compound 344,265 indicates increased electronic responsiveness while still preserving sufficient stability. This balance facilitates efficient noncovalent interactions with the target protein while maintaining adequate kinetic stability of the molecule.

These findings suggest that the electronic properties of the identified compound are suitable for effective interaction with TEM *β*-lactamase, supporting its potential as a promising inhibitor. Since protein–ligand binding in this system is predominantly governed by noncovalent interactions, intermolecular forces and structural complementarity play key roles in complex formation.

**FIGURE 11:**
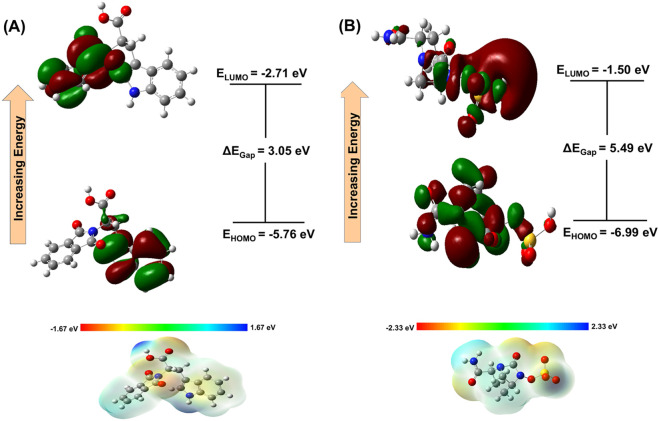
Frontier molecular orbitals (HOMO-LUMO) and molecular electrostatic potential maps. **(A)** 344265, **(B)** Avibactam.

Additionally, various global reactivity descriptors derived from HOMO and LUMO energies were calculated (Table 4). The molecular electrostatic potential (MEP) surface (Figure 11) further illustrates the charge distribution within the molecule, with regions of high electron density shown in red, electron-deficient regions in blue, and intermediate electrostatic potential in green to yellow.

**TABLE 4:** Molecular descriptors derived from DFT analysis.

Compound code	HOMO	LUMO	Energy gap (ΔE)	Ionization potential (I)	Electron affinity (A)	Electro negativity (χ)	Chemical potential (µ)	Global hardness (η)	Softness (Ѕ)	Electrophilicity (ω)
344,265	−5.76	−2.71	3.05	5.76	2.71	4.23	−4.23	1.52	0.32	5.88
Avibactam	−7.01	−1.52	5.49	7.01	1.52	4.27	−4.27	2.75	0.18	3.31

## 4 Discussion

*E. coli* is a relevant Gram-negative pathogen that causes a wide spectrum of infections, including UTIs, bloodstream infections, and intra-abdominal infections, and has a heavy healthcare burden across the globe (Ron, 2010). The rise in MDR *E. coli* cases, especially due to the development of *β*-lactamases that degrade *β*-lactam antibiotics, has severely restricted treatment options and led to increased morbidity and mortality (Pitout and Laupland, 2008). *β*-lactamase inhibitors, namely, clavulanic acid, sulbactam, and tazobactam, are combined with *β*-lactam antibiotics to inhibit enzymatic hydrolysis and thereby restore antibacterial efficacy (González-Bello et al., 2020). However, resistance to these molecules has been increasingly reported in recent studies (Drawz and Bonomo, 2010). Although avibactam is a clinically effective modern *β*-lactamase inhibitor with activity against a broad range of class A enzymes, including TEM types, potential adverse effects and therapeutic limitations highlight the importance of exploring alternative inhibitory molecules (Cheng et al., 2020; Torres et al., 2023; George et al., 2025).

Avibactam was selected as the reference inhibitor for comparative analysis due to its well-characterized inhibitory mechanism and broad-spectrum activity against class A *β*-lactamases, including TEM variants. As a non-*β*-lactam diazabicyclooctane (DBO) inhibitor, avibactam exhibits a reversible covalent mode of inhibition and enhanced stability against hydrolysis compared with classical *β*-lactam-based inhibitors (Drawz and Bonomo, 2010; Ehmann et al., 2012; Lahiri et al., 2014). These properties, together with their established clinical use, make it a stringent and reliable benchmark for evaluating novel inhibitors in structure-based computational studies.

Other advanced DBO inhibitors, such as relebactam and nacubactam, share the same core mechanism of reversible covalent inhibition and enhanced stability, differing primarily in their inhibitory spectra and pharmacological profiles. Relebactam functions as a *β*-lactamase inhibitor in combination with imipenem, whereas nacubactam also exhibits affinity for PBP2, contributing to its dual-functional profile (Papp-Wallace et al., 2018; Mallalieu et al., 2020). Given the conserved DBO scaffold and shared mode of enzyme inhibition, avibactam serves as a representative advanced-generation inhibitor for benchmarking. Therefore, comparative evaluation against avibactam provides a meaningful, mechanistically consistent basis for assessing the relative binding affinity and interaction profiles of the identified compounds.

Recent computational studies have explored new molecules targeting TEM *β*-lactamases; for instance, phytochemicals derived from *Mangifera indica* have been screened against TEM-1 to identify potential inhibitors (Isa and Geidam, 2026). Considering the global dissemination and sustained clinical relevance of TEM *β*-lactamases, exploring chemically diverse compounds remains important for supporting durable therapeutic efficacy.

More recent progress has shown that ML methods have the potential to discover promising lead compounds against a wide range of biological targets (Dara et al., 2022). In this study, the performance of different classifiers was evaluated using several metrics, including kappa statistic, sensitivity, specificity, accuracy, and ROC analysis (Fawcett, 2006; Asha Kiranmai and Jaya Laxmi, 2018). Among the tested models, Random Forest showed the best performance with the highest kappa value (0.76) and high predictive accuracy, indicating strong agreement between predicted and actual classifications. The high ROC value was an additional indicator of the model’s strength in discriminating between active and inactive compounds. Sensitivity and specificity analyses also demonstrated the superior ability of Random Forest to correctly identify positive instances, whereas the PART classifier showed comparatively lower sensitivity. In general, Random Forest was found to be more effective than the other classifiers, followed by J48 and PART. This optimized model was used to screen 4681 antibacterial compounds, identifying 3576 active candidates. Further selection of these compounds was based on pharmacokinetic and toxicity properties, and 55 molecules were identified that met the selection criteria. The virtual screening of these candidates against TEM-1 and TEM-235 was then performed based on binding affinity. All non-toxic compounds and the reference inhibitor, avibactam, were selected for further docking analysis. This confirms that the compound has a moderate number.

Comparative evaluation of binding energies across both TEM variants ultimately led to the finding of three candidate compounds. These compounds formed stable interactions with the target proteins’ active-site residues, including H-bonds and hydrophobic contacts, which possibly contributed to their enhanced binding affinity.

In this study, MD simulations gave a detailed understanding of the structural dynamics and conformational behavior of the complexes (Durrant and McCammon, 2011). RMSD was calculated for the protein Cα atoms along with all ligand atoms to assess structural deviations during the simulation (Maruyama et al., 2023). The analysis indicated that compound 344,265 (2-(1,3-dioxo-1,3-dihydro-2H-isoindol-2-yl)-3-(1H-indol-3-yl) propanoic acid) formed the most stable complexes with both TEM-1 and TEM-235 throughout the trajectory. The compound is a small molecule with a molecular formula of C_19_H_14_N_2_O_4_ and a molecular weight of 334.3 g/mol, and moderate lipophilicity (XLogP3 = 2.6), indicating a balanced solubility-permeability profile. It contains 2 hydrogen-bond donors and 4 hydrogen-bond acceptors, thereby facilitating stable protein-ligand interactions. The TPSA value of 90.5 Å^2^ suggests favorable oral bioavailability. Additionally, the presence of 4 rotatable bonds indicates moderate conformational flexibility, while its neutral charge contributes to stable binding interactions. Moreover, the compound showed no evidence of hepatotoxicity, cardiotoxicity, carcinogenicity, or mutagenicity, indicating a favorable safety profile.

The reduced RMSF fluctuations, strong interaction energies, and increased structural compactness observed for this compound with both TEM variants suggest its potential as an effective inhibitor. Notably, the screened compound exhibited lower RMSD values compared with the reference inhibitor avibactam, indicating enhanced conformational stability. RMSF analysis of the Cα atoms of TEM was performed to evaluate the flexibility of individual amino acid residues, where higher RMSF values designate increased flexibility and lower values reflect substantial structural stability (Jamroz et al., 2012). The TEM-1_344,265 and TEM-235_344,265 complexes exhibited comparatively lower RMSF values, indicating enhanced structural stability. In contrast, the avibactam-bound TEM-1 and TEM-235 complexes exhibited relatively higher RMSF values, suggesting moderate stabilization of the protein structure. The protein-ligand complexes also exhibited strong Lennard-Jones short-range interactions, with high average interaction energies, reflecting stable binding. Overall, the MD trajectories revealed that in spite of local conformational variations, the compound conserved stable interactions with key active-site residues over time.

Binding free energy was estimated using the MM/PBSA approach, which integrates molecular mechanics energy with the Poisson–Boltzmann electrostatic solvation model (Genheden and Ryde, 2015). This method is widely applied to rank and prioritize screened compounds based on their binding energetics. In the present study, compound 344,265 exhibited negative binding free energies against all TEM variants, supporting the docking results and indicating favorable binding affinity. Overall, the cumulative analyses suggested that the lead compound exhibited stronger binding characteristics than the reference inhibitor, avibactam.

PCA was implemented to investigate the influence of inhibitor association on the target protein’s conformational dynamics and structural variations. In the two-dimensional projection plot, complexes occupying smaller phase-space regions represent more stable conformational clusters, whereas broader distributions indicate higher structural flexibility (Buslaev et al., 2016; Buslaev et al., 2016; Buslaev et al., 2016). The results showed that the TEM-1_344,265 and TEM-235_344,265 complexes occupied comparatively smaller conformational spaces than the other complexes, suggesting greater structural stability. This reduced conformational flexibility may be attributed to optimized ligand–protein interactions that limit the backbone motions of the protein. The 344,265-bound complexes consistently exhibited well-defined low-energy basins in the FEL, indicating the presence of stable conformational clusters throughout the simulations (Tse et al., 2020; Tse et al., 2020). Furthermore, the Gibbs free energy values across the complexes remained within a stable range (0–13.7 kJ/mol), supporting the thermodynamic stability of the observed conformational states. DCCM analysis was performed to evaluate the influence of inhibitor binding on protein motions, with positive correlations indicating coordinated movements and negative correlations indicating anticorrelated motions (Yu, 2020; Yu, 2020). In this analysis, 344265-bound systems exhibit reduced anticorrelated fluctuations and more localized correlated motion, suggesting improved coordination of residue dynamics. These findings suggest that compound 344265 enhances the dynamic stability of both TEM variants compared with the reference inhibitor, avibactam.

Finally, the DFT analysis indicates that compound 344265 has a balanced electronic profile, as reflected in its HOMO–LUMO energy gap of 3.05 eV, indicating an optimal balance between kinetic stability and chemical reactivity. Such a balance is essential for bioactive molecules, as it enables sufficient electronic adaptability to support effective noncovalent interactions while maintaining structural integrity under physiological conditions (Kumar et al., 2024). The comparatively lower energy gap suggests enhanced electronic responsiveness, which may facilitate charge redistribution and strengthen molecular recognition during protein–ligand binding. In contrast, the higher energy gap (5.49 eV) observed for avibactam reflects greater intrinsic stability but relatively lower electronic flexibility. Furthermore, the MEP analysis demonstrates a well-defined distribution of electron-rich and electron-deficient regions, supporting the formation of favorable intermolecular interactions (Akbari et al., 2024). Overall, these electronic characteristics complement the docking and molecular dynamics findings, reinforcing the potential of compound 344,265 as a promising inhibitor of TEM *β*-lactamase.

Therefore, the *in silico* analyses suggest that 344,265 exhibits promising *β*-lactamase-inhibitory potential. However, further experimental validation through *in vitro* studies is essential to confirm its efficacy and establish its potential as a therapeutic candidate.

## 5 Limitation

In this study, a descriptor-based ML approach was employed, retaining all calculated molecular features to preserve comprehensive chemical information. While this enables broad representation of molecular characteristics, the absence of explicit feature selection or dimensionality reduction may limit the prioritization of optimal features and the interpretability of the model. Although the employed algorithms can accommodate a large number of input variables, integrating systematic feature selection strategies could further enhance computational efficiency and predictive performance.

The present study provides computational evidence supporting the inhibitory potential of the identified compounds against TEM β-lactamase; however, experimental validation is required to establish their translational and clinical relevance. The antibacterial activity of these compounds should be confirmed through *in vitro* assays, particularly minimum inhibitory concentration (MIC) testing against resistant strains, to quantify their efficacy and validate their mechanism of action.

Future investigations can focus on detailed characterization of protein–ligand interactions using advanced structural and biophysical techniques to gain deeper insight into binding mechanisms. In addition, rational optimization of the chemical scaffolds may improve binding affinity, specificity, and pharmacological properties. Targeting multiple resistance pathways simultaneously may further reduce the likelihood of resistance development, thereby advancing effective therapeutic strategies against MDR bacterial infections.

## 6 Conclusion

The ML-guided virtual screening of antibacterial compounds identified potential *β*-lactamase inhibitors against TEM-1 and TEM-235 of MDR *E. coli*. Among the screened compounds, 344265 exhibited a favorable pharmacokinetic profile, superior interaction, stability, binding affinity, and chemical stability based on ADMET analyses, molecular docking, MD simulations, binding free energy calculations, and DFT analyses. Although these are promising computational results, *in vitro* assays are essential to confirm their inhibitory activity and to facilitate further optimization for future therapeutic development.

## Data Availability

The original contributions presented in the study are included in the article/Supplementary Material, further inquiries can be directed to the corresponding author.
